# Engineering Tools for Regulating Hypoxia in Tumour Models

**DOI:** 10.1111/jcmm.16759

**Published:** 2021-07-02

**Authors:** Min Hee Kim, Steven D. Green, Chien‐Chi Lin, Heiko Konig

**Affiliations:** ^1^ Department of Biomedical Engineering Indiana University‐Purdue University Indianapolis Indianapolis IN US; ^2^ Department of Medicine Division of Hematology/Oncology Indiana University School of Medicine Indianapolis IN US; ^3^ Indiana University Melvin and Bren Simon Comprehensive Cancer Center Indianapolis IN US

## Abstract

Major advances in the field of genomic technologies have led to an improvement in cancer diagnosis, classification and prognostication. However, many cancers remain incurable due to the development of drug resistance, minimal residual disease (MRD) and disease relapse, highlighting an incomplete understanding of the mechanisms underlying these processes. In recent years, the impact of non‐genetic factors on neoplastic transformations has increasingly been acknowledged, and growing evidence suggests that low oxygen (O_2_) levels (ie hypoxia) in the tumour microenvironment play a critical role in the development and treatment of cancer. As a result, there is a growing need to develop research tools capable of reproducing physiologically relevant O_2_ conditions encountered by cancer cells in their natural environments in order to gain in‐depth insight into tumour cell metabolism and function. In this review, the authors highlight the importance of hypoxia in the pathogenesis of malignant diseases and provide an overview of novel engineering tools that have the potential to further drive this evolving, yet technically challenging, field of cancer research.

## 1 INTRODUCTION

**FIGURE 1:**
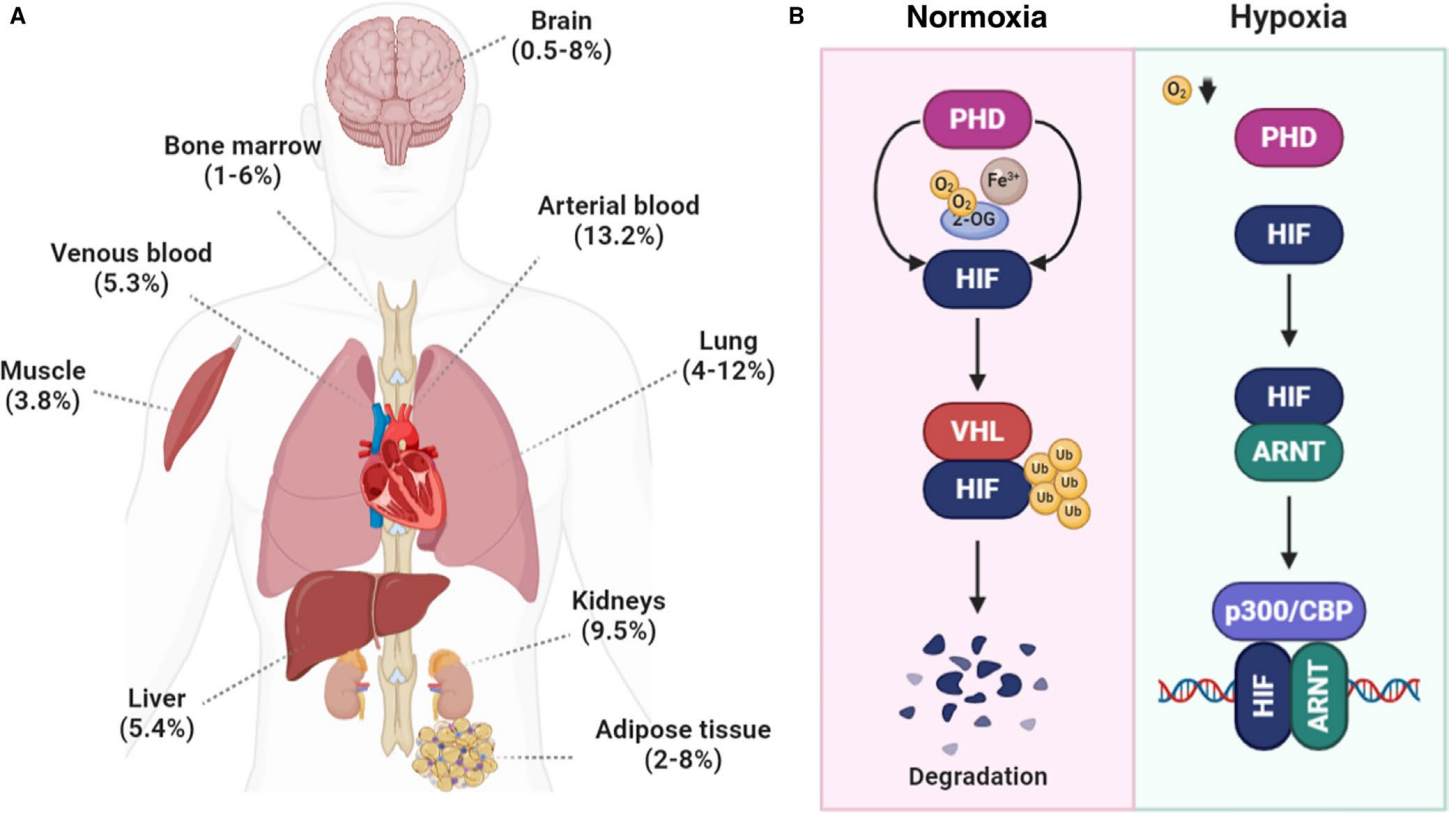
Tissue oxygenation and HIF regulation. (A) O_2_ concentrations as measured in selected mammalian tissues. (B) Regulation of HIF stability by O_2_

Oxygen (O_2_) tension in the body varies greatly, depending on the location and the physiological condition of the specific tissue.^1^ The level of tissue oxygenation plays a critical role in both healthy and diseased physiological processes, such as ischaemia, tumours and inflammation.^2^ In healthy tissues, O_2_ concentration drops from 20% in the lungs to ~13% in the alveoli, and ~5% in the circulation.^3^ The O_2_ content in multicellular structures can further decrease to below 5%. Other tissue‐specific O_2_ levels include 5% in the venous blood, 1%‐7% in the bone marrow, 0.5%‐7% in the brain and 1% in the cartilage (**Figure** **1A**).^4^, ^5^ Increasing lines of evidence suggest that hypoxia is an innate facet of cancer, as the proliferation of malignant cells quickly exceeds the diffusion limit of O_2_ (100‐200 µm) resulting in inadequate oxygenation.^6^ The vascular, metabolic and oncogenic adaptations that ensue are known to be critical to the biology of various cancers. Representing a therapeutic liability, tumour hypoxia is increasingly being explored for the development of personalized treatment approaches to influence tumour growth, metastatic potential and drug resistance. However, hypoxia‐targeted strategies have only yielded limited success to date. Part of this unsatisfactory outcome may be attributed to the lack of appropriate experimental methods, which often involve the manipulation and study of cells exposed to non‐physiologic O_2_ concentrations and gradients that poorly reflect the physiologic conditions encountered by tumour cells in their natural environments. Hence, generating hypoxic conditions and hypoxic gradients in the in vitro setting has received increasing attention because hypoxia is capable of inducing, via hypoxia‐inducible factor α (HIF‐1α), a host of cell survival responses (eg autophagy).^7^ In this review, we highlight several fundamental concepts of hypoxia, its metabolic adaptation and impact on tumour biology. We also discuss the need and recent progress of novel engineering tools and methodologies required to generate hypoxia and O_2_ gradients, which are needed to further drive progress in this emerging field of research.

## 2 HYPOXIA‐INDUCIBLE FACTORS

The transcription factor hypoxia‐inducible factor (HIF‐1) is a key mediator for transmitting changes in O_2_ tension into changes in genetic transcription allowing for cellular adaptation.^8^, ^9^, ^10^ The level of HIF‐1 ultimately regulates the expression of a wide range of adaptive processes, including the conversion from oxidative to glycolytic metabolism and angiogenesis.^11^ Structurally, HIF‐1 is a heterodimeric complex comprised of a stable beta subunit and O_2_‐sensitive alpha subunits. Under normoxic conditions, prolyl hydroxylases (PHD) hydroxylate the alpha subunits of HIF, leading to ubiquitylation by the von Hippel Lindau (VHL) complex and subsequent proteasomal degradation (**Figure** **1B**).^12^, ^13^, ^14^ Factor inhibiting HIF (FIH) also hydroxylates an asparagine residue of HIF‐1α when O_2_ is available, blocking its interaction with the transcriptional coactivator protein p300 and preventing transactivation of certain HIF target genes.^15^, ^16^ Hypoxia inactivates PHD and FIH, resulting in the accumulation of HIF‐1 and its translocation to the nucleus where it interacts with HIF‐1β and binds to hypoxia‐response elements.^17^, ^18^ Notably, these regulatory mechanisms are also affected by the severity and duration of hypoxia.^19^, ^20^ In addition, it has been shown that HIF‐1 is stabilized by an acidic intracellular pH, which often develops as a result of hypoxic metabolic changes.^21^ Beyond being functionally important for the adaptation of normal and malignant cells to hypoxic conditions, HIF has been implicated in promoting genetic instability, immune evasion, migration and metastasis and stem cell maintenance.^22^, ^23^, ^24^, ^25^ Accordingly, elevated levels of HIF‐1 have been demonstrated in some studies to be an independent negative prognostic indicator portending increased risk of metastasis, mortality and other adverse features in a variety of cancers including breast, lung and pancreas.^26^, ^27^ There is also evidence that HIF interacts with key tumour suppressor and proto‐oncoproteins such as p53 and MYC.^28^, ^29^ However, for a minority of cancers, such as cervical cancer for example, it does not appear to have any prognostic significance.^30^, ^31^

## 3 METABOLIC ADAPTATIONS TO HYPOXIA

When O_2_ availability decreases, cellular metabolism shifts from oxidative phosphorylation to the less efficient glycolysis. To maintain this process, pyruvate oxidizes NADH and is reduced to lactate via lactate dehydrogenase (LDH). As lactate accumulates, the cytoplasm becomes increasingly acidic which inhibits glycolysis, so lactate is excreted from cells by monocarboxylate transporters.^32^ HIF‐1 up‐regulates the production of many glycolytic genes including isozymes of LDH that favour pyruvate reduction, lactate transporters and multiple other enzymes including hexokinase 1 and 3, aldolase A and C and pyruvate dehydrogenase kinase 1.^33^, ^34^, ^35^, ^36^ HIF‐1 also up‐regulates COX4‐2, a subunit of complex IV of the electron transport chain which appears to be more efficient under hypoxic conditions and potentially generates less reactive oxygen species (ROS).^37^, ^38^ When the demand for intracellular glucose increases, cancer cells can utilize glycogen to remain viable and proliferate.^39^ This too limits the production of ROS, avoiding senescence.^40^ Hypoxic cells can also utilize glutamine via both oxidative metabolism and reductive carboxylation.^41^, ^42^, ^43^ Finally, in addition to glucose, glycogen and glutamine, there is evidence that hypoxic cancer cells may use other carbon sources such as exogenous acetate to produce acetyl‐CoA, and perhaps nutrients released from organelles as a consequence of autophagy.^44^, ^45^ Furthermore, it has been shown that there is metabolic interplay between hypoxic and normoxic tumour regions. For example, tumour vascular endothelial cells have been noted to be highly glycolytic, thus allowing more O_2_ to reach further into the tumour.^44^ Another study has shown that a symbiotic relationship can exist between normoxic tumour regions that oxidize lactate to spare glucose and hypoxic tumour regions which metabolize glucose into lactate, thus providing a metabolic substrate for the normoxic regions.^45^

## 4 THE ROLE OF HYPOXIA IN SOLID MALIGNANCIEs

In solid cancers, hypoxic tumour cells respond by producing angiogenic factors, but this pathologically induced process yields new vessels that are structurally and functionally suboptimal compared with vessels produced by well‐coordinated physiologic angiogenesis.^6^ Chaotic non‐laminar blood flow, leakiness, and vascular remodelling lead to dynamic changes in O_2_ delivery, with hypoxia lasting from seconds to days or of a cyclical nature.^46^ The result of the high metabolic demands of malignant cells combined with limited O_2_ delivery due to abnormal vasculature is that even highly vascularized cancers or tumour regions can contain areas of severe hypoxia.^11^ Similarly, regardless of the degree of hypoxia, the pO_2_ level of a tumour is always lower than corresponding normal tissue, resulting in hypoxia relative to physioxia.

Hypoxic conditions lead to elevated genomic instability, the selection of cells that have diminished DNA repair (down‐regulated *MLH1*, *MSH2*, *RAD51*) and apoptotic potential (*TP53* mutations), and a dampening of the antitumour immune response.^47^, ^48^, ^49^, ^50^, ^51^ It also leads to the development of protective stem cell niches and enhanced expression of multidrug resistance proteins.^52^, ^53^, ^54^ Furthermore, the lower rate of proliferation of hypoxic cancer cells decreases the effectiveness of cytotoxic chemotherapeutics that work best in actively dividing cells. Finally, in addition to resistance to chemotherapy and radiotherapy, hypoxia has been shown to contribute to resistance to immunotherapy via a variety of mechanisms including down‐regulation of MHC‐I and up‐regulation of immune checkpoints.^55^, ^56^ The net result of which is that low O_2_ levels in solid cancers can generate a more mutagenic and treatment‐resistant phenotype. As a result, tumour hypoxia has been linked to unfavourable cancer outcomes. In prostate cancer, for example, hypoxia has been associated with biochemical relapse independent of factors such as Gleason score, prostate‐specific antigen (PSA) levels or T‐category.^57^ Another study found an association between HIF‐1 and vascular endothelial growth factor (VEGF) expression on diagnostic tumour biopsies and biochemical relapse following radiotherapy or radical prostatectomy, although it has been acknowledged that factors unrelated to hypoxia may up‐regulate HIF‐1.^58^ Patients with head and neck cancer treated with radiation alone were found to have an association between tumour hypoxia, as measured with electrodes, and inferior overall survival (OS) and higher rates of local recurrence.^59^, ^60^ Studies have also shown that hypoxia seems to increase the propensity for metastatic disease across multiple cancer types, such as cervical and breast cancer.^61^, ^62^, ^63^ A limitation of the available clinical data is that a variety of different techniques were used to measure hypoxia, each with their attendant advantages and disadvantages, which have been reviewed elsewhere.^2^

There have been various attempts to therapeutically exploit hypoxia as a differentiating metabolic characteristic of malignant cells. These have included radiosensitizers, antiangiogenics and hypoxia‐activated pro‐drugs amongst others. For example, one approach involving a combination of accelerated radiotherapy, the inhalation of carbogen (98% O_2_ and 2% CO_2_) and the vasoactive compound nicotinamide (ARCON) was compared with accelerated radiotherapy alone in a phase III trial of patients with laryngeal cancer.^64^ The combined approach resulted in a statistically significant improvement in regional control, albeit without improvement in local control. Another phase II trial of radiation, carbogen and nicotinamide compared to radiation alone in patients with locally advanced bladder cancer resulted in a significant improvement in OS and local relapse rates with the hypoxia‐directed treatment.^65^ An example of a radiosensitizer that has been studied is nimorazole, which was tested in combination with radiation in a phase III trial of patients with supraglottic laryngeal and pharyngeal cancers versus placebo and radiation, and demonstrated improved locoregional control.^66^ However, a number of hypoxia‐activated pro‐drugs demonstrating promising early activity in phase I and II trials ultimately led to negative phase III trials. These include tirapazamine in head and neck cancer and evofosfamide (TH‐302) in advanced pancreatic cancer and soft tissue sarcomas.^67^, ^68^

## 5 THE MOLECULAR HALLMARKS OF HYPOXIA

One major challenge in directly measuring the extent of hypoxia in malignant tissues is the significant amount of both intratumoral heterogeneity and intertumoral heterogeneity in O_2_ status for each cancer type, which can change over time. There has therefore been a growing effort to understand the molecular hallmarks of hypoxia, ultimately using diagnostic tumour biopsies as both an indirect reflection of the broader O_2_ microenvironment over time and to deduce a given tumour's dependence on hypoxia for its proliferation. To this end, single‐nucleotide variants (SNVs) and copy number aberrations (CNAs) of *TP53*, *MYC* and *PTEN* have consistently been associated with hypoxia in multiple cancer types.^69^ There was, however, a notable degree of variation in SNV hypoxia signatures between tumour types, which emphasizes the need for further, in‐depth studies in each malignancy. Another study identified a genomic signature of the metabolic shift associated with tumour hypoxia across multiple cancer types.^70^ In addition to protein and mRNA, microRNA expression has been associated with hypoxia.^71^, ^72^ For example, miR‐210 abundance was associated with hypoxia across 18 tumour types in one study, although more studies are needed to determine their precise regulatory role.^69^ Some limitations of the clinical utility of hypoxia biomarkers include a degree of dependence on adequate sampling of the tumour to account for special heterogeneity and that many markers are regulated by both hypoxia‐dependent and hypoxia‐independent mechanisms.

It is becoming increasingly clear that microenvironmental pressures such as hypoxia may be shaping the mutational architecture of cancer, selecting for subclones with aggressive features.^69^ A major challenge is to identify those tumours with a “hypoxic driver” molecular or genetic phenotype in which hypoxia is a primary driver of the cancer's behaviour, as this subgroup will be enriched in predictive value for response to hypoxia‐targeted treatments.^73^ For example, a retrospective analysis of the aforementioned nimorazole trial examined a 15‐gene hypoxia panel in pre‐treatment biopsies and found that only patients with hypoxic tumours as determined by the panel had improved local control and survival.^74^ It has also been hypothesized that hypoxic niches in tissues such as the bone marrow may provide shelter to cancer stem cells and are at least partially responsible for treatment resistance in leukaemia and other diseases.^75^ Therefore, a greater understanding of the key molecular pathways underpinning hypoxic cancer cells’ resistance to treatments may promote the development of novel targets and therapies. However, a major reason why a significant knowledge gap persists in our understanding of the role of hypoxia is the difficulty of studying cancers ex vivo, which often involves the use of un‐physiologic cell culture techniques carried out in ambient air or in chambers that maintain a constant level of hypoxia. Therefore, progress in our understanding of the molecular characteristics of hypoxia, as well as its therapeutic exploitation, will likely require a tandem progress in experimental models.

## 6 PATHOPHYSIOLOGICAL EFFECTS OF OXYGEN GRADIENTS

Increasing lines of evidence suggest that O_2_ gradients might play an important role in the process of drug resistance and cancer cell survival, potentially by providing “escape routes” along which neoplastic cells migrate when a cell death signal is activated by cytotoxic therapy. In fact, cellular migration along gradients, including chemokine,^76^ cytokine^77^ or growth factor^78^ gradients, has long been recognized as a fundamental process in cellular adaption. Based on previously reported computer simulation data by Cristini *et al,* which indicated that tumour cells follow O_2_ concentration gradients, Mosadegh and colleagues utilized three dimensional paper‐based invasion assays to investigate whether gradients of O_2_ direct tumour cell migration.^79^, ^80^ Using the human adenocarcinoma cell line A549 and three independently derived cell lines, the authors observed that fractions of tumour cells undergo chemotaxis towards higher levels of O_2_, concluding that migratory responses to O_2_ gradients might represent a distinctive feature to identify cellular subgroups within complex populations.^80^ In line with these findings, Lin et al demonstrated that cervical cancer cells migrate faster and over longer distances compared with human umbilical cord vein endothelial cells under hypoxic conditions in a microfluidic cell co‐culturing system device.^81^ Similarly, but in contrast to the observations in A549 cells, Sleeboom et al described that breast cancer cells and their respective cancer stem cells migrate towards low O_2_ regions in a microfluidic gradient device.^82^ Ceradini and colleagues reported that in the process of tissue repair and regeneration, CXCR4^+^ stem/progenitor cell recruitment to injured tissues is mediated by SDF‐1 and hypoxic gradients. Taking into account that i.) the CXCR4/SDF‐1 axis has previously been shown to play a critical role in tumour cell trafficking in a broad range of malignancies and ii.) neoplastic states are frequently characterized by hypoxic conditions, tumour‐associated microenvironments might utilize the same mechanisms to recruit circulating cancer cells to O_2_‐deprived niches, thereby potentially providing a sanctuary to facilitate the development of drug resistance and disease relapse.^83^, ^84^ Intriguingly, the chemotactic migration of leukemic cancer cells was significantly enhanced when treated with doxorubicin and daunorubicin in a microfluidic microcirculation mimetic device.^85^ Overall, the concept of O_2_ gradient‐directed migration is highly relevant from a clinical and translational standpoint as current strategies to decrease tumour vascularity augment tumour hypoxia with the associated risk of promoting tumour cell survival. To date, the detailed mechanisms involved in O_2_‐directed migration remain to be elucidated. As a deeper understanding of these processes will likely open new avenues in cancer therapy, novel technologies providing O_2_ gradients in vitro are urgently needed.

## 7 HYPOXIA‐INDUCING CHEMICALS

Intracellular hypoxia‐like responses can be created or mimicked by using chemical reagents, such as sodium sulphite (Na_2_SO_3_), cobalt chloride (CoCl_2_) and the iron chelator desferrioxamine (DFO) (**Table** **1**). Sodium sulphite serves as a O_2_ scavenging agent by forming sodium sulphate (Na_2_SO_4_), resulting in hypoxic conditions (~20 mmHg) after 20‐30 minutes.^86^, ^87^ Bhatti *et al* used sodium sulphite anoxic solution to generate low O_2_ tension (lower than 20 mmHg) for culturing human dermal neonatal fibroblasts.^88^ On the other hand, CoCl_2_ and DFO induce hypoxia‐like responses in the cells by blocking the degradation and thus accumulation of intracellular HIF‐1α.^89^ Mechanistically, CoCl_2_ reacts with O_2_ to form a CoO compound, generating a hypoxia‐like intracellular environment and inhibiting the PHD pathway. CoCl_2_ acts by either chelating the iron core of HIF‐1α and replacing it with cobalt or taking up the VHL‐binding domain of HIF‐1α, thus rescuing it from degradation.^90^ Heirani‐Tabasi *et al* explored the effect of hypoxia‐mimicking agents such as CoCl_2_ and DFO on several signalling molecules that are involved in migration of adipose‐derived mesenchymal stem cells (Ad‐MSCs) in vitro. On the other hand, DFO inhibits the PHD pathway through chelating iron in the media. DFO could increase both cell migration and expression of genes such as VEGF‐A, VEGF‐C, MAPK4, INPP4B and IL‐8.^91^ Various studies have exploited this mechanism for inducing HIF‐mediated responses.^92^, ^93^ Alternatively, inhibition of PHD through oxoglutarate analogues such as dimethyloxalyglycine (DMOG) can inhibit the hydroxylation of HIF‐1α similar to culturing cells under hypoxic conditions (12.5%‐2.5%).^94^ While adding chemical reagents to induce hypoxia or mimic intracellular hypoxic response is convenient, the potential cytotoxicity and unintended cellular behaviours (eg cell division and morphology) may occur due to the added chemicals.^95^, ^96^

**TABLE 1:** Chemical reactions of hypoxia‐mimicking agents

Hypoxia‐mimicking agent	Chemical reaction	References
Sodium sulphite (Na_2_SO_3_)	2 Na_2_SO_3_ + O_2_ → 2 Na_2_SO_4_	^84^
Cobalt chloride (CoCl_2_)	2 CoCl_2_ + O_2_ → 2 CoO + 2 Cl_2_	86, 87
Desferrioxamine	C_25_H_48_N_6_O_8_ + Fe^3+^ → Chelating compound	87, 118
Zinc chloride (ZnCl_2_)	2 ZnCl_2_ + O_2_ → 2 ZnO + 2 Cl_2_	^119^

## 8 ENGINEERING TOOLS FOR CREATING HYPOXIA

### 8.1 Hypoxic chambers

Various engineering tools are available for controlling O_2_ content for in vitro cell culture and related experiments, including the bulky glove box and hypoxia workstations. A simple plastic chamber system with controllable gas inets may also be used to create a uniform O_2_ content within the enclosed chamber, which fits in a conventional incubator (1%‐10%).^97^ Furthermore, gas cylinders, which usually contain a desired gas mixture, are needed to produce and maintain the conditions of physical hypoxia in the chamber (0.0‐1.5 mg/L, control group showed 7.5 mg/L level of oxygen).^98^, ^99^ However, this system cannot reach low levels of hypoxia due to the large volume of air to be exchanged. In addition, O_2_ concentration would increase drastically even after a short exposure to ambient air for media change, which may significantly influence hypoxia‐related gene expression.^95^, ^100^ One common shortfall of glove boxes, hypoxia workstations and simple hypoxic chambers is that only one specific level of O_2_ content can be maintained at any given time, hencing limiting the study of hypoxia‐related cellular responses to a fixed O_2_ content.

### 8.2 Microfluidic devices

**FIGURE 2:**
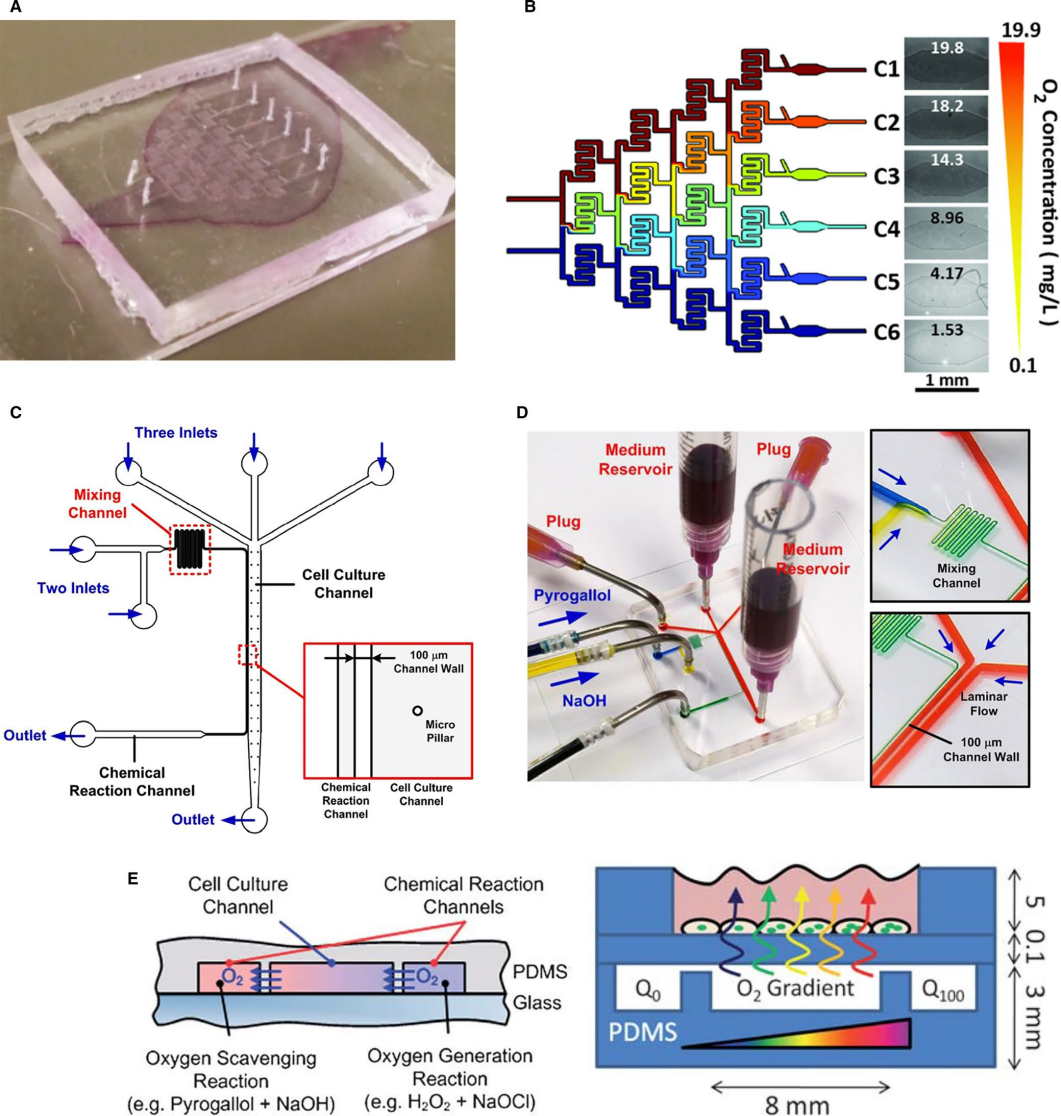
Microfluidic‐based engineering tools for controlling O_2_ content. (A) Image of a gradient‐generating microfluidic device. (B) Schematic of step O_2_ concentrations formed in each outlet of the gradient‐generating microfluidic device. O_2_ diffusion in the chamber is controlled by diffusion through 3‐sided glass coating. (C) Schematic of the microfluidic device capable of performing collective cell migration assays with O_2_ gradients. (D) Photographs of the fabricated microfluidic devices. O_2_ scavenging chemical reaction between pyrogallol and NaOH was performed in the reaction channel in order to generate O_2_ gradient. (E) Schematic illustration of O_2_ consumption and gradient‐generating mechanism in a microfluidic chamber. Reprint with permission

To circumvent the disadvantages of hypoxic chamber systems, other sophisticated engineering systems have been developed to generate complex hypoxia patterns for in vitro cell culture, such as microfluidic devices. Microfluidic platforms enable precise controls over the local microenvironmental properties, including flow rate and physicochemical compositions of the media, including O_2_ content. In general, microfluidic devices are fabricated from polydimethylsiloxane (PDMS) or polystyrene (PS).^101^ In microfluidic devices, the culture area geometry and flow path can be precisely controlled to permit real‐time imaging of hypoxic cell culture. It is also possible to create complex hypoxia patterns within microfluidic devices.^102^ To generate O_2_ gradients inside the microfluidic devices, several methods have been employed, including (i) introducing O_2_ scavenging agents into the devices and (ii) controlling O_2_ concentration through gas supply channels. Khan *et al* designed complex microfluidic chambers using an established soft lithography procedure.^102^ After manufacturing the chamber, the interior of the chamber was coated with 3‐sided glass to control the permeability of O_2_. Using this method, O_2_ gradients of various spatial resolutions (from 0.1 to 19.9 mg/L) can be rapidly and conveniently established (**Figure** **2A,B**) through adjusting the flow rate of medium pre‐equilibrated with lower oxygen tension.^95^ Shih *et al* showed that the spatially confined chemical reaction could generate stable O_2_ gradients within the microfluidic device (21% O_2_ nomaxia and 1% O_2_ hypoxia).^101^ The O_2_ scavenging chemical reaction between pyrogallol (benzene‐1,2,3‐triol) and NaOH occurred in the chemical reaction chamber (**Figure** **2C,D**). When pyrogallol is added in alkaline solution, it absorbs O_2_ rapidly and creates a “sink” that induces a unidirectional diffusion of O_2_ to generate an O_2_ gradient (**Figure** **2E**). It is possible to alter the range and steepness of the gradient O_2_ in the same device by changing the composition of the gas mixture fed into the culture areas with different sizes and shapes.^103^ The disadvantages of microfluidic systems include complicated manufacturing processes, the need of flow control instruments and device set‐up. In addition, it is not suitable for long‐term or large‐scale cell studies.^104^

### 8.3 Enzymatic reactions

**FIGURE 3:**
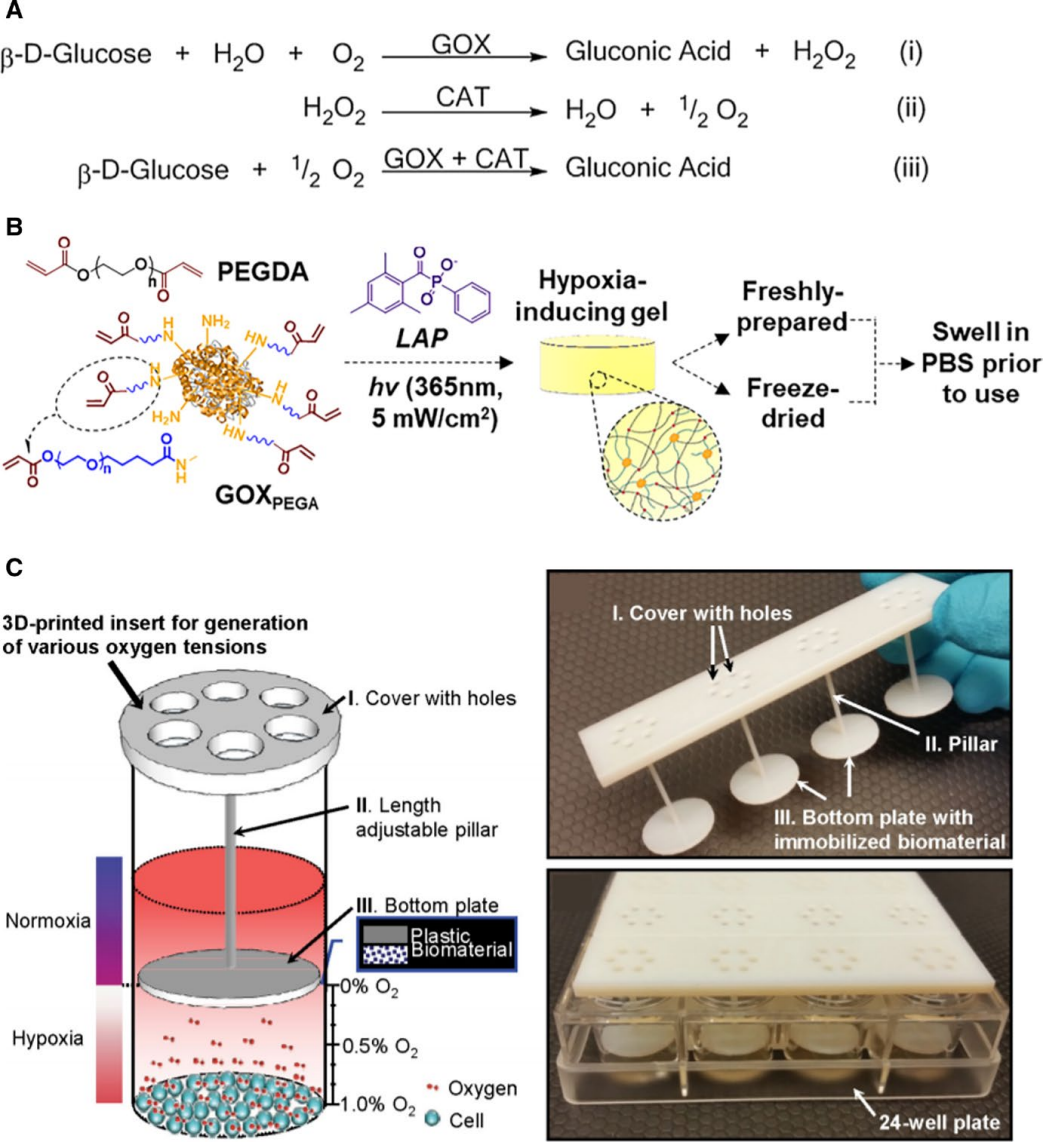
O_2_‐consuming enzymes for controlling O_2_ content. (A) The principle of the glucose oxidase/catalase (GOX/CAT) system for independent control of hypoxia and H_2_O_2_ level. (B) Reaction scheme of GOX immobilized PEGDA hydrogel. (C) Conceptual illustration and digital images of the 3D‐printed insert with immobilized biomaterial for on demand generation of various O_2_ tensions for in vitro cell cultures. The biomaterial consisting of glucose oxidase and catalase enzymes consumes O_2_ in the cell culture media without interfering with the testing environment. Reprint with permission

Recently, O_2_‐consuming enzymes have been exploited as an alternative strategy to create hypoxic culture environments.^105^ The most widely used O_2_‐consuming enzyme is glucose oxidase (GOX), which converts glucose, oxygen and water into gluconic acid and hydrogen peroxide (H_2_O_2_).^106^ An endogenous enzyme, GOX, has been used in cancer diagnosis and treatment. For example, the consumption of glucose and oxygen may be exploited for cancer‐starvation and hypoxia‐activated therapy, respectively.^107^ On the other hand, the reaction product gluconic acid may be employed for pH‐responsive drug release. Finally, H_2_O_2_ generated in the reaction can be converted into toxic hydroxyl radicals for cancer cell killing.^107^ While the reaction of GOX is fast and effective, one significant drawback for its application in cell studies is the production of cytotoxic H_2_O_2_, the accumulation of which can lead to undesired cellular toxicity, but can also inactivate GOX.^108^, ^109^ To minimize the cytotoxic by‐product of GOX reactions, catalase (CAT) can be used to reduce H_2_O_2_ into water. However, this reaction partially offsets hypoxia by producing half an oxygen (**Figure** **3A**). Dawes *et al* designed GOX immobilized polyethylene glycol diacrylate (PEGDA) hydrogel for extended hypoxic cell cultures (**Figure** **3B**).^105^ Immobilization of O_2_‐consuming GOX within covalently cross‐linked hydrogels provides an easy method to control solution O_2_ tension without using external devices (2.5%‐9%). Furthermore, through the introduction of CAT in cell culture media, duration of hypoxic conditions and concentrations of H_2_O_2_ were adjusted to minimize cytotoxicity and enzyme inactivation (decreased from 9 mM to 2 mM). Hudson *et al* followed up the study with improved processing methods to increase the flexibility and stability of the hypoxia‐inducing hydrogel system.^110^ While both freshly prepared and lyophilized PEGDA‐GOX hydrogel generated low O_2_ environments rapidly, lyophilization negatively affected enzyme activity. This could be prevented by using cryoprotectants, such as trehalose and raffinose, during freeze‐drying. Ideally, this approach would not only increase the flexibility of using the enzyme‐immobilized hydrogels but also add commercialization potential. In another experiment, Li *et al* designed a new approach for O_2_ tensions using GOX.^111^ Specifically, GOX/CAT‐containing chitosan coating was applied to the 3D‐printed inserts (**Figure** **3C**). Since O_2_‐consuming biomaterials were immobilized in the chitosan matrix, O_2_ consumption only occurs on the surface of biomaterial and hypoxia formed underneath the polymer/enzyme coating (4.7 mmHg to 61.1 mmHg).

**FIGURE 4:**
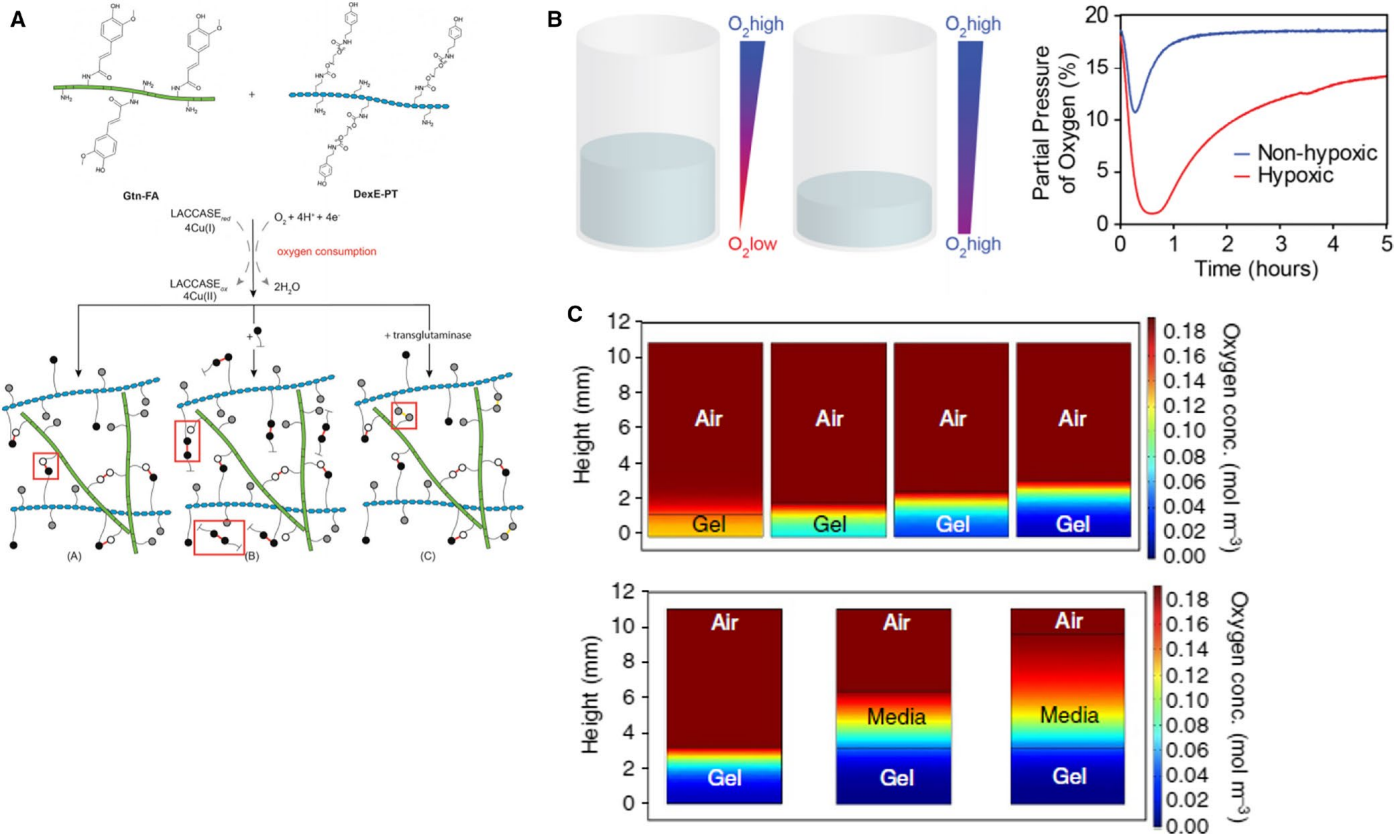
Laccase‐based hypoxia‐inducible hydrogels for controlling O_2_ content in 3D. (A) Schematic representation of HI hydrogel formation. HI hydrogels are formed via laccase‐mediated dimerization of FA molecules with O_2_ consumption. (B) Hydrogel height controls O_2_ gradients. (C) Model predictions of O_2_ levels and gradients after 30 min of hydrogel formation in the layer model (airgel and air–media‐gel). Reprint with permission

Another enzyme‐mediated O_2_‐consuming reaction is through using laccase,^112^ which also consumes O_2_ during its catalytic reaction. Park *et al* developed hypoxia‐inducible (HI) hydrogels by immobilizing laccase substrates (eg ferulic acid [FA] or tyramine [TA]) to the polymer chains. Laccase is a Cu‐containing enzyme that catalyses one‐electron phenolic compounds by transferring four electrons from four substrate molecules to one molecule of molecular oxygen that is then reduced to water.^113^ Specifically, FA or TA was first immobilized to the polymers (eg gelatin and dextran) via standard carbodiimide chemistry.^112^, ^114^ The addition of laccase to the FA/TA‐immobilized polymers resulted in enzymatic cross‐linking of the polymers while the reactions consumed O_2_ simultaneously (1.8%‐15% according to the thickness of hydrogel). This HI hydrogel technique was used to create a hypoxic microenvironment for a variety of cellular in vitro studies, with potential options in in vivo settings (**Figure** **4A**).^115^, ^116^ The duration of hypoxia can be extended by increasing the thickness of the HI hydrogels. As the thickness of the hydrogel increases, the diffusion of O_2_ in the media or atmosphere decreases and the hypoxic duration in the matrix increases (**Figure** **4B,C**). Laccase‐mediated reactions were shown to be cytocompatible, and the HI hydrogels were supportive of vascular network formation from the encapsulated endothelial colony‐forming cells (ECFCs) owing to the increased secretion of angiogenic growth factors. In principle, this strategy can be easily applied to other natural/synthetic macromers, such as polyethylene glycol (PEG) or hyaluronic acid (HA). Some drawbacks of this approach include the following: (1) the extent of hypoxia is limited by the amount of substrate immobilized to the polymer, and (2) the maintenance of hypoxia relies on limiting diffusion of O_2_ into the hydrogel network.

### 8.4 Diffusion barriers

O_2_ gradients can be also created and adjusted using a diffusion barrior with different gas permeability. For example, Yi *et al* designed human glioblastoma (GBM) on a chip model using gas‐permeable polydimethylsiloxane (PDMS) as the diffusion barrior.^117^ Using a 3D‐printing system, the chip was first fabricated on a non‐permeable glass substrate, followed by printing a ring of endothelial cell‐ladened brain decellularized extracellular matrix (BdECM). Finally, BdECM bioink ladened with GBM cells was printed inside the ring, while the top of the silicon chamber was covered with a glass slip. In this design, O_2_ was only available to the cells *via* the gas‐permeable silicone chamber wall. As the cells located in the centre of the device consumed O_2_, a radial O_2_ gradient was generated. The combination of cell‐laden matrix with a mechanism for generating O_2_ gradient using 3D bioprinting provides a novel approach to interrogate the influence of biochemical and biophysical cues on cancer cell progression.

In another study, Campillo *et al* designed custom‐made co‐culture system based on thin membranes permeable to O_2_.^118^ The device consisted of two PDMS well layers separated by a commercially available membrane. O_2_ concentration over the cell culture can be tightly controlled via direct diffusion from 12% to 1% through the gas‐permeable membrane from a gas source connected to the lower layer. Therefore, cells cultured in the Transwell insert were exposed to either the same or different O_2_ levels as those cells growing on the chip surface. Another approach to generating O_2_ gradients through a diffusion barrier is using layered papers.^80^ For example, Derda *et al* reported a strategy for controlling the distribution of cultured cells in 3D by fabricating multi‐laminated structures of fibre‐supported hydrogels with each layer composed of paper impregnated with an ECM hydrogel.^119^ In each layer, Matrigel precursor containing suspended cells was added to a paper support. By stacking and destacking cell‐containing layers, it was possible to manipulate gradients of nutrients and O_2_ in these constructs and to characterize cells grown in these complex gradients. These diffusion barrier systems are a powerful tool to precisely mimic and control the O_2_ content in the in vitro cancer models. However, one common disadvantage of diffusion barrier induced O_2_ gradients is that the process for creating the barriers (eg printing and stacking of paper‐based barrier, printing of silicone chambers) maybe complicated and time‐consuming.

## 9 Conclusion

Cancer continues to represent a leading cause of mortality on a global scale, accounting for approximately 20 million new cases and 10 million deaths each year.^120^ Despite progress in understanding cancer biology, current treatment strategies generally fail to achieve a cure due to the development of treatment resistance and disease relapse. Growing evidence suggests that the poor outcome is at least in part due to a small fraction of cancer cells (minimal residual disease, MRD) that outlive initial treatments by migrating into specialized, frequently O_2_‐deprived, niches where they seek protection from therapeutic elimination.^121^, ^122^ As a result, tumour hypoxia has moved into the centre of interest as a therapeutically exploitable phenomenon in a wide variety of neoplasms. Efforts to target the multifaceted complex mechanisms underlying MRD, however, have only yielded limited success so far.^123^, ^124^, ^125^ A major contributor to the slow progress in targeting MRD is the difficulty of studying hypoxic cancer cells ex vivo and the use of un‐physiologic cell culture techniques. While O_2_ levels can vary widely within the human body, it is estimated that most tissues range between 2% and 9% O_2_. In contrast, rapidly growing, aggressive tumour tissue can be nearly anoxic, particularly in the centre.^126^, ^127^ Standard cell culturing techniques carried out in ambient air (normoxia, 21% O_2_) are thus not consistent with physiological conditions. Current efforts to study cancer cell biology under physiological, hypoxic conditions in vitro utilize specialized incubators and hypoxic chambers to control the O_2_ tension in cell culture assays. These approaches, however, are technically cumbersome and limited by O_2_ diffusion and equilibration. In addition, the use of hypoxic incubators and chambers foresees constant levels of hypoxia, whereas recent studies, including mathematical modelling of O_2_ distribution in tumour tissues, suggest the existence of O_2_ gradients.^80^, ^128^, ^129^ In order to develop novel strategies to more effectively target cancer cells, a deeper understanding of the molecular mechanisms induced by different levels of hypoxia is needed. This, however, requires innovative, cancer‐relevant technologies to examine therapeutic escape mechanisms that focus on the role of hypoxia and O_2_ gradients within the tumour tissue. Such technologies may hold the key for the identification and delineation of yet unknown mechanisms underlying drug resistance and MRD, opening new research horizons for the development of novel therapeutic targets and strategies. Yet, the development of experimental methods and tools to study tumour cell biology under controlled O_2_ conditions has been challenging. As reviewed here, recent efforts with microfluidic devices, enzymatic reactions, hydrogels and 3D‐printing platforms represent innovative solutions to overcome previous limitations and offer opportunities to mimic physiologically relevant O_2_ dynamics and spatial properties such as encountered by neoplastic cells in their natural habitats. It can be expected that such engineered platforms will help unveil formerly unknown key molecular pathways that act in response to hypoxic stress and O_2_ gradients. Such new knowledge will likely improve our ability to differentiate and target the metabolism of cancer cells while sparing normal tissues, thereby enhancing the chances of a cure.

## CONFLICT OF INTEREST

The authors confirm that there are no conflicts of interest.

## AUTHOR CONTRIBUTIONS

**Min Hee Kim:** Writing‐original draft (equal); Writing‐review & editing (equal). **Steven D Green:** Writing‐original draft (equal); Writing‐review & editing (equal). **Chien‐Chi Lin:** Writing‐original draft (equal); Writing‐review & editing (equal). **Heiko Konig:** Writing‐original draft (equal); Writing‐review & editing (equal).

## Data Availability

Data sharing is not applicable to this article as no new data were created or analyzed in this study.
